# Imaging pheochromocytoma in small animals: preclinical models to improve diagnosis and treatment

**DOI:** 10.1186/s13550-021-00855-x

**Published:** 2021-12-11

**Authors:** Hermine Mohr, Alessia Foscarini, Katja Steiger, Simone Ballke, Christoph Rischpler, Franz Schilling, Natalia S. Pellegata

**Affiliations:** 1 Institute for Diabetes and Cancer, Helmholtz Zentrum München, Ingolstaedter Landstrasse 1, 85764 Neuherberg, Germany; 2 Joint Heidelberg-IDC Translational Diabetes Program, Heidelberg University Hospital, Heidelberg, Germany; 3 Institute of Pathology, School of Medicine, Technical University of Munich, Munich, Germany; 4 Department of Nuclear Medicine, School of Medicine, Technical University of Munich, Munich, Germany; 5 Department of Biology and Biotechnology “L. Spallanzani”, University of Pavia, Pavia, Italy

**Keywords:** Positron emission tomography, Paraganglioma, ^18^F-LMI1195, ^68^GA-DOTATATE, Xenograft, MSOT, Norepinephrine transporter, MENX-affected rats

## Abstract

Pheochromocytomas (PCCs) and paragangliomas (PGLs), together referred to as PPGLs, are rare chromaffin cell-derived tumors. They require timely diagnosis as this is the only way to achieve a cure through surgery and because of the potentially serious cardiovascular complications and sometimes life-threatening comorbidities that can occur if left untreated. The biochemical diagnosis of PPGLs has improved over the last decades, and the knowledge of the underlying genetics has dramatically increased. In addition to conventional anatomical imaging by CT and MRI for PPGL detection, new functional imaging modalities have emerged as very useful for patient surveillance and stratification for therapy. The availability of validated and predictive animal models of cancer is essential for translating molecular, imaging and therapy response findings from the bench to the bedside. This is especially true for rare tumors, such as PPGLs, for which access to large cohorts of patients is limited. There are few animal models of PPGLs that have been instrumental in refining imaging modalities for early tumor detection, as well as in identifying and evaluating novel imaging tracers holding promise for the detection and/or treatment of human PPGLs. The in vivo PPGL models mainly include xenografts/allografts generated by engrafting rat or mouse cell lines, as no representative human cell line is available. In addition, there is a model of endogenous PCCs (i.e., MENX rats) that was characterized in our laboratory. In this review, we will summarize the contribution that various representative models of PPGL have given to the visualization of these tumors in vivo and we present an example of a tracer first evaluated in MENX rats, and then translated to the detection of these tumors in human patients. In addition, we will illustrate briefly the potential of ex vivo biological imaging of intact adrenal glands in MENX rats.

## Background

Pheochromocytomas (PCCs) and paragangliomas (PGLs) are catecholamine-secreting tumors arising from the transformation of neural crest-derived chromaffin cells located in the adrenal medulla or in paravertebral autonomic ganglia, respectively. These tumors are together referred to as PPGLs. The first-line and sole curative treatment for PPGLs is surgery, but subsequent recurrence or metastatic spread always remains possible. For inoperable metastatic disease, there is no approved medical therapy in Europe, although molecular targeted therapies are currently being investigated. For slow-progressing PPGLs, radionuclide therapy using [^177^Lu]-1,4,7,10-tetraazacyclododecane-tetraacetic acid (DOTA)-Tyr3-octreotide (DOTATATE) or [^131^I]-Meta-iodobenzylguanidine (MIBG) is the preferred first-line therapy, with the latter being the only approved therapy in the USA [1]. However, the former treatment only works if tumors express somatostatin receptor 2 (SSTR2), and the latter has variable efficacy as outlines below. For rapidly progressing metastatic PPGLs, chemotherapy is the recommended first-line option but has shown limited success.

PPGLs cause unspecific symptoms in patients, and this can hamper their timely diagnosis. Hypertension, headaches, palpitation and diaphoresis are most often reported [2]. However, patients can also present with anxiety, nausea, new onset of diabetes, weakness, pallor, flushing, abdominal chest pains or orthostatic hypotension. Yet, none of these symptoms alone clearly points to the presence of PPGLs, hence, this disease gained the title “the great mimic” [3]. The lack of specific symptoms, combined with the fact that these tumors are uncommon, makes it difficult for clinicians to suspect PPGLS as the underlying cause. Given the severe comorbidities associated with undiagnosed/untreated PPGLs, it is important to develop sensitive and specific tools for their early diagnosis.

Chromaffin cells naturally produce norepinephrine, epinephrine and dopamine. In PPGL patients, the levels of these catecholamines are, with some exceptions, strongly enhanced. Based on recent guidelines, PPGLs are initially diagnosed by the assessment of elevated levels of catecholamines or their metabolites (metanephrines), being the measurement of plasma-free or urinary fractioned metanephrines the most reliable method [4]. While norepinephrine- and epinephrine-overproducing tumors can be reliably identified by the detection of metanephrines, the much rarer dopamine-producing PPGLs need an additional test assessing the increase in circulating levels of methoxytyramine, the O-methylated metabolite of dopamine [5]. A positive biochemical diagnosis of PPGL is usually followed by localization of the tumor using imaging techniques, such as computed tomography (CT), T2-weighted magnetic resonance imaging (MRI), [^123^I]-MIBG scintigraphy and, more recently, by functional positron emission tomography (PET) imaging [6] (see below).

### Subtypes of PPGL

Over 30–40% of PPGL patients carry germline mutations in at least 25 cancer susceptibility genes, and additional 35–40% of cases associate with somatic driver mutations, so that about 70% of PPGLs is genetically defined. The underlying mutations associate with specific gene expression signatures. Based on these signatures, PPGLs were classified into three main subtypeswere classified into three main subtypes: a pseudohypoxic, a kinase signaling and a Wnt-altered cluster. These clusters are associated with specific genetic mutations, catecholamine secretion profiles and a variable risk of metastases [7]. The pseudohypoxic cluster comprises tumors having mutations in, e.g., *SDHA*, *SDHB*, *SDHD*, *VHL* and *EPAS1* genes, and typically consists of norepinephrine- and dopamine-producing tumors. PPGLs of the pseudohypoxic cluster have a higher risk to metastasize than those belonging to the other clusters. Mutations in, e.g., *NF1*, *RET*, *TMEM127* and *HRAS* cause PPGLs belonging to the kinase signaling cluster and having mostly an adrenergic biochemical phenotype. PPGLs of this subtype are usually benign, but show a high degree of recurrence and tumor multiplicity [8]. The most recently identified subcluster is the Wnt-altered one, for which only sporadic mutations in *CSED1* and *MAML3* have been described as the underlying genetic cause. The Wnt-altered cluster displays a mixed biochemical profile, having both adrenergic and noradrenergic phenotypes. Based on the TCGA study on the molecular characterization of PPGLs, the most extensive to date, pseudohypoxic tumors account for over 30% of cases, those with a kinase activation profile for over 20% and those in the Wnt subcluster for less than 10% [7].

### Diagnosis of PPGL by anatomical and functional imaging

While it has been estimated that PPGLs are the underlying cause in 0.1 to 1% of all hypertensive patients, about 25% of all PPGL are incidentally discovered during imaging studies for unrelated disorders [9]. Moreover, in 0.05–0.1% of autopsy examinations, PPGLs were detected that were not diagnosed during the lifetime of the diseased persons [10]. It needs to be considered that undiagnosed PPGLs can carry relevant morbidity, can cause significant comorbidities, may lead to fatal complications during anesthesia (preformed to treat other disorders) or can cause the fatal shock syndrome. In contrast, the prognosis for PPGLs that are detected at an early stage is very good, thereby supporting the need for tools that allow a timely diagnosis and accurate staging of these tumors.

**Fig. 1:**
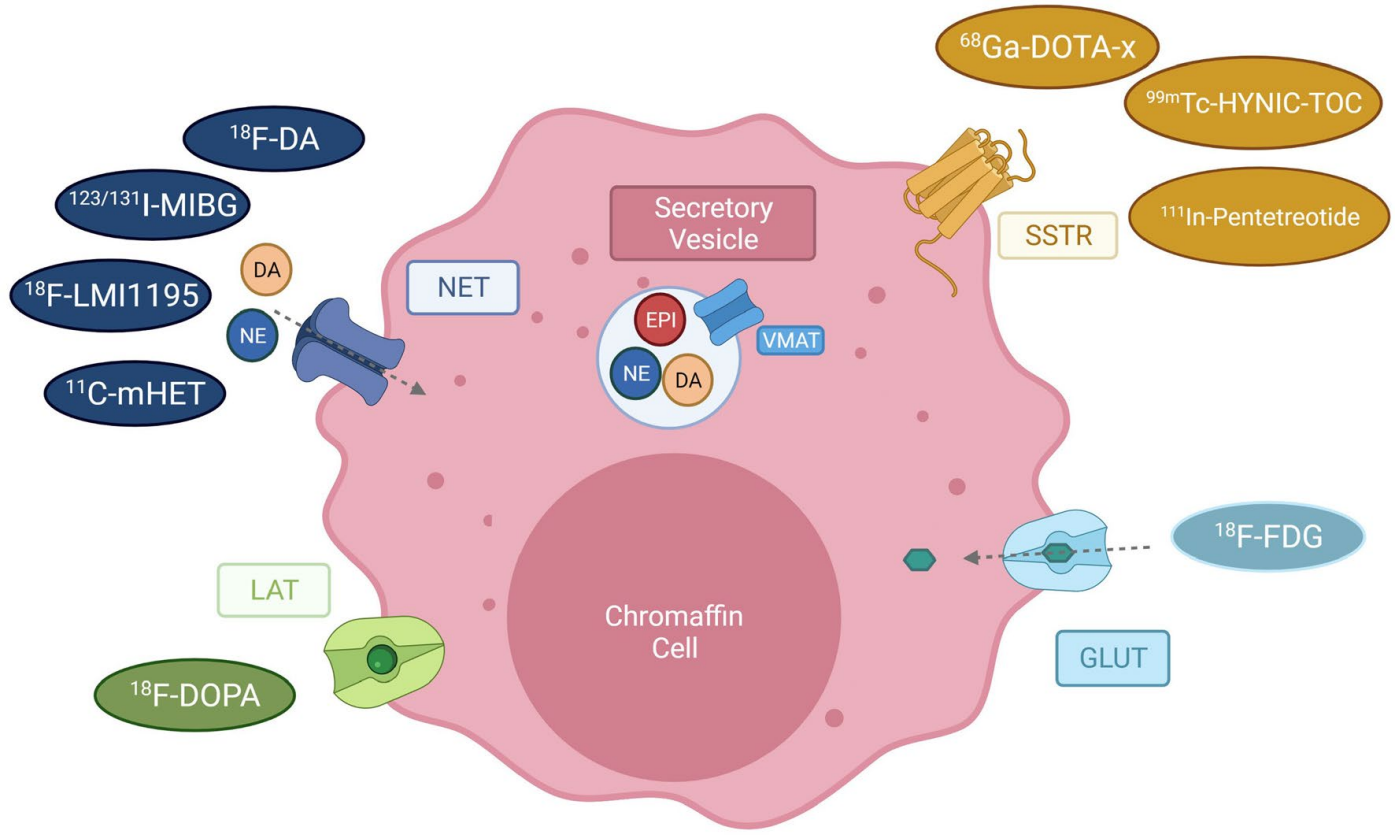
Available PET tracers for imaging of pheochromocytoma and paraganglioma. Depicted are receptors of chromaffin cells used as targets for PET imaging (*LAT* large amino acid transporter, *NET* Norepinephrine transporter, *SSTR* Somatostatin receptor, GLUT = glucose transporter) and their tracers ([^18^F]-DOPA = [^18^F]-dihydroxyphenylalanine; [^68^Ga]-DOTA-X = derivatives of [^68^Ga]1,4,7,10-tetraazacyclododecane-tetraacetic acid, e.g., DOTATATE, DOTA-NOC, DOTA-TOC, [^99m^Tc] -HYNIC-TOC = [^99m^Tc]-hydrazinonicotinyl-Tyr3-octreotide, [^18^F]-FDG = , [^18^F]-Fluordesoxyglucose). The vesicular monoamine transporter (VMAT) transports catecholamines as norepinephrine (NE), epinephrine (EPI) and dopamine (DA) into secretory vesicles, from where they are released into the extracellular matrix. Reuptake or norepinephrine is mediated via the NET transporter. ( Adapted from Blanchet et al., 2011 [82])

Although biochemical tests have high predictive value for the presence of PPGLs, so that subtype, size and even tumor location (adrenal, extra-adrenal) of the primary tumor may be inferred from these data [5], further validation by imaging modalities is recommended [11]. Anatomical imaging, such as CT or MRI, should be considered as the first choice to identify the location of the tumor, as indicated in the recent European Association of Nuclear Medicine (EANM) guidelines for imaging PPGLs [12]. Precise knowledge of the tumor location is necessary to guide surgery, the only curative treatment for most PPGLs. However, the diagnosis of PPGLs by anatomical imaging can be very challenging. Indeed, benign pathological changes in the adrenal glands may be misdiagnosed as tumors [13]. Substantial improvements in the diagnosis of PPGLs could be obtained by combining anatomical with functional imaging, hence several modalities have been developed that take advantage of the expression of specific receptors or transport systems in chromaffin cells. Depending on genetic risk factors, molecular cluster, size and location of the original tumor, additional functional imaging scans or postsurgical follow-up screens are recommended [4]. The current functional imaging tracers available for PPGLs can be grouped in four major classes depending on the targeted processes/molecules: (1) catecholamine metabolism/norepinephrine transporter expression; (2) somatostatin receptors (SSTRs); (3) glucose uptake; and (4) amino acid uptake (Fig. 1). Up to recently, the gold standard imaging approach to detect PPGLs was scintigraphy with [^123^I]-MIBG a norepinephrine analog, which is taken up by chromaffin cells via the norepinephrine transport (NET) system [14]. Comparison among tumors belonging to the various clusters, as well as of benign *versus* metastatic PPGLs, revealed varying sensitivities toward the individual tracers [15]. Therefore, beyond localization, the imaging signature obtained by functional imaging can help in cluster-specific patients stratification in view of subsequent intervention by targeted therapy or radionuclide therapy [16]. Given that the various PPGL subtypes show different sensitivities to the various radionuclides depending on their characteristics (mutations, location, aggressiveness), an algorithm has been proposed for the use of nuclear imaging of these tumors [12]. Being SDHx-associated PPGLs more prone to metastasize than other molecular subtypes, the identification of sensitive and specific tracers for these pseudohypoxic tumors would be clinically relevant.

**Fig. 2:**
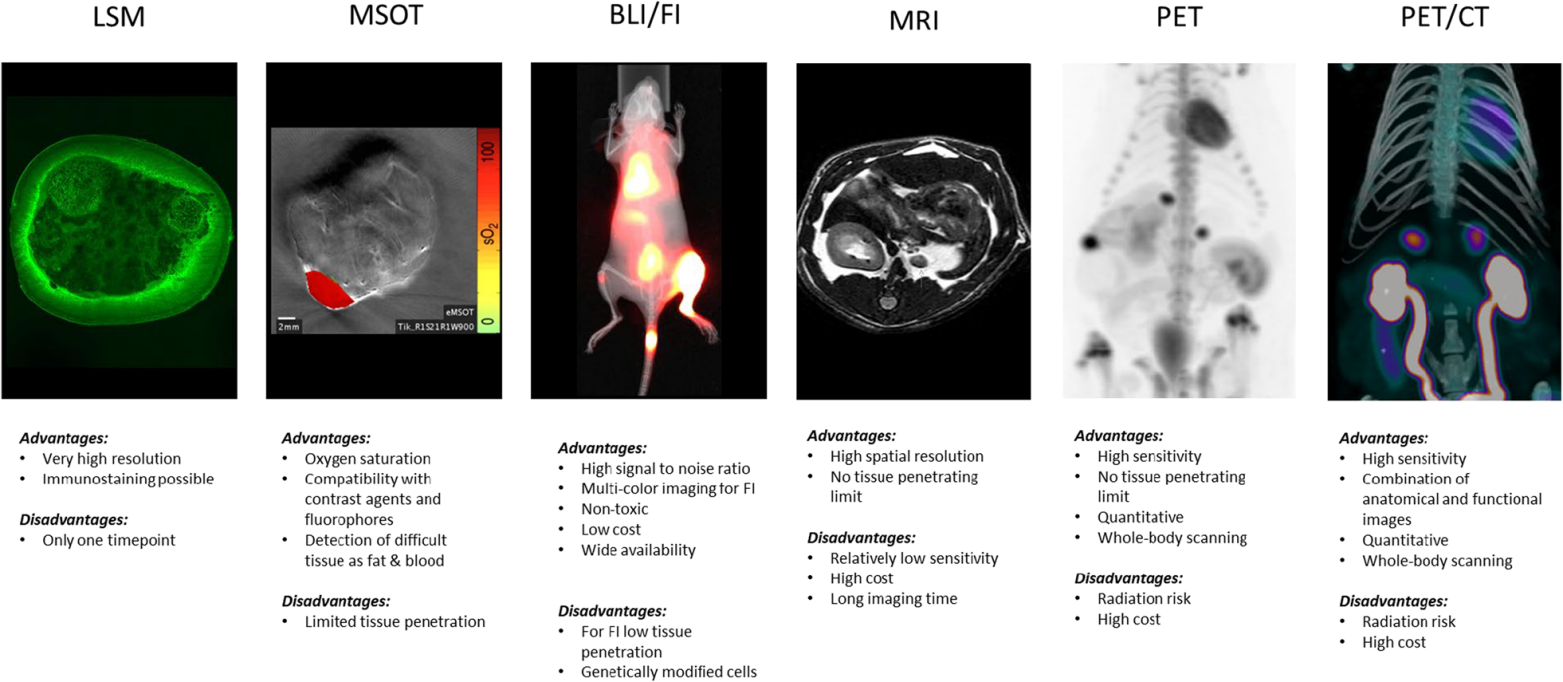
Image modalities used for PPGL research in small animals. *LSM* Light sheet microscopy (PCC from a mut/mut MENX rat), *MSOT* multispectral optoacoustic tomography (MENX PCC senograft), *BLI*/*FI* bioluminescence/fluorescence imaging (metastasized MPC-luc cells in an allograft model, kindly provided by Martin Ullrich), *PET* positron emission tomography (MENX rat with [^18^F]-LMI1195), *PET*/*CT* combined picture of PET and computed tomography (MENX rat with [^18^F]-LMI1195 and CT). Advantages and disadvantage of each modality are listed

Reliable detection of small primary tumors, of metastases, and prediction of tumor characteristics are urgently needed to improve prognosis and to achieve tailored treatments for PPGL patients. Animal models of cancer are crucial for the evaluation and development of novel imaging modalities. This is especially true for rare tumors for which access to large cohorts of patients to evaluate novel medical imaging or treatment approaches is highly limited. In this review, we will present the contribution that studies on animal models have provided to PPGL detection. We will focus on the different imaging tools that have been tested with the aim of validating the suitability of the animal model, of improving tumor detection and monitoring therapy response, of assessing imaging tracer distribution as well as improving or developing image-guided therapy for PPGLs (Fig. 2).

### Imaging of xenograft and allograft PPGL models

Owing to the paucity of genetically engineered mouse strains that reproduce the human disease [17], most in vivo studies so far conducted relied on the injection of PPGLs cell lines into immunocompromised mice. Given that establishing human tumor cell lines has proved to be an arduous task, there is currently none available that represents a functional catecholamine-secreting PPGL. The few sporadically reported human PPGL cell lines were never distributed to the research community or display a progenitor cell phenotype. The most commonly used rodent cell lines are PC12 and MPC cells. PC12 is a clonal line originating from PCC cells derived from the tumor of an irradiated rat [18], which were further passaged subcutaneously in rats [19]. PC12 cells are noradrenergic, synthesizing and storing dopamine and norepinephrine [19]. PC12 represent an extensively used model in neuroscience research as these cells differentiate into neurons when exposed to nerve growth factor (NGF) [20]. MPC (mouse pheochromocytoma cells) cells, and their aggressive derivative MTT (mouse tumor tissue) cells, were derived from a PCC that developed in a *Nf1* knockout mouse [21, 22]. MPC cells mainly secrete epinephrine and strongly express the receptor tyrosine kinase, Ret and the GDNF receptor, GFRalpha1 [23], indicating a progenitor-like genetic signature.

Xenografts/allograft models have been generated by engrafting these rodent PPGL cell lines into mice. PC12-derived xenografts were used to study the efficacy of both [^131^I]-MIBG radionuclide therapy [24] and the receptor tyrosine kinase inhibitors sunitinib and sorafenib [25]. Despite being effective against this tumor model, neither ^131I^MIBG [26] nor sunitinib [27] therapy caused the clinical benefits expected based on the outcomes in these preclinical studies, indicating that more clinically relevant models should be employed. Allografts generated by injecting MPC cells have proved to be a very valuable experimental tool to evaluate novel imaging modalities for PPGL diagnosis and theranostic approaches. Studies with patient-derived xenografts (PDXs) aimed at propagating this precious material in vivo are still at their early stages, and the groups attempting to establish these models have reported low engraftment rates [28, 29]. Therefore, the potential of PDXs to contribute to PPGL research is still to be determined.

### Anatomical imaging

Anatomical imaging gathers information about the basic physical structure and the composition of a tissue by measuring characteristics such as electron density for CT or proton density for MRI. CT was the first imaging modality to offer detailed 3D imaging of internal anatomy [30] using serial radiographic projections of X-rays to create a cross-sectional image. In the practical clinical guidelines of the endocrine society on the “Diagnosis and Treatment of Pheochromocytoma and Paraganglioma,” CT is recommended as initial imaging modality [11]. In case the tumor is located in the adrenal gland, anatomical imaging can be considered sufficient for the initial diagnosis, for extra-adrenal PPGL additional functional imaging is recommended by the EANM Guidelines [12].

#### MicroCT in PPGL research

As the detection of metastatic PPGLs is a highly clinically relevant issue, studies have focused on the identification of sensitive imaging modalities allowing to visualize metastases in animal models. Megapixel charge-coupled device detectors and significant increases in computer memory and speed, allowed to adapt traditional clinical CT to small animals, a technique called microCT, which offers high spatial resolution [31]. The first PPGL metastatic model was established in 2006 by Ohta et al*.* by injecting MPC intravenously into the tail vein of nude mice [32]. The cells were able to colonize the body and form liver tumors, but only rarely in other organs (lungs, bone, adrenal glands). Given that the liver is among the preferred sites of PPGL metastases, this model might give useful information in view of clinical translation. MicroCT using the hepatobiliary-specific contrast agent glycery1-2-oley-1,3-bis-[7-(3-amino-2,4,6-triiodophenyl)-heptanoate] (DHOG) allowed detection of tumors as small as 0.35 mm, thereby establishing this imaging modality as a promising technique to visualize small metastases in live animals. A caveat of this model is the fast growth of the tumors and the rapidly deteriorating health of the mice.

#### MRI for PPGL research

CT is more often used in clinical practice to detect and locate PPGLs due to lower costs, higher availability and better spatial resolution. However, MRI reaches higher sensitivity for detection of PGLs or metastasized PPGLs, and being free of ionizing radiation is therefore better suited for pregnant women, children or patients with adverse reactions to an iodinated contrast medium [33]. Moreover, it has been suggested that MRI is superior to CT in characterizing human adrenal masses [34]. A study in mice compared the use of the contrast-enhanced microCT technique versus non-enhanced MRI for the detection of metastasizing MPC cells [35]. Herein, both methods were equally suited to detect liver lesions. MRI was superior to microCT in detecting lesions at other organs (lung, adrenal, ovarian and renal), whereas neither modality detected early bone lesions. The authors suggested that it might be more suitable to use MRI instead of CT to lower the radiation burden to the animals.

### Functional imaging

#### Scintigraphy with [^123/131^I]-MIBG and derivatives

**Fig. 3:**
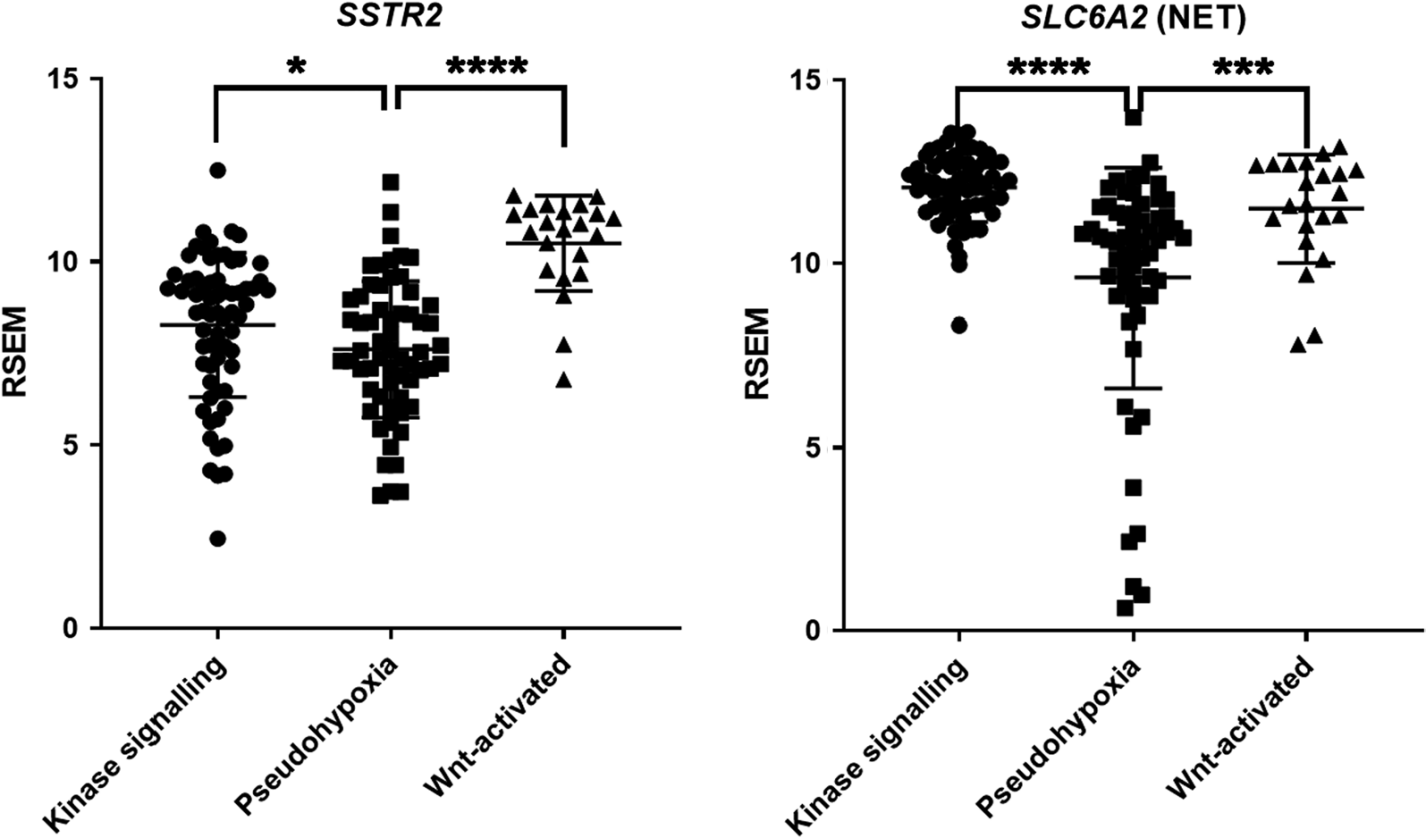
Expression of Somatostatin and NET transporter in TCGA database. Expression levels of the *SSTR2* and *SLC6A2* (encoding for NET) in the PPGL TCGA dataset RSEM-normalized, exported via www.cbioportal.org (patient stratification into clusters according to Fishbein et al. [6]. Low level of SSTR2 expression in some patients belonging to the pseudohypoxia cluster

[^123^I]-MIBG was the first tracer to be developed for PPGLs, and until recently planar [^123^I]-MIBG scintigraphy was the gold standard in PPGL imaging [36], although it has now been replaced by single-photon emission computer tomography (SPECT) allowing more accurate anatomical localization. MIBG is a structural analogue of norepinephrine, and it is taken up by the NET present on the chromaffin cells’ membrane (uptake 1) and stored inside the secretory vesicles (Fig. 1). The signal of the tracer is proportional to vesicular granules density inside the tumor cells [37]. Given that MIBG uptake scanning enables whole-body imaging, thereby allowing the detection of extra-adrenal tumors and metastatic deposits, it is often the preferred initial screening modality. MIBG can be labeled with either ^131^I or ^123^I, the latter being more suitable for imaging (especially when using SPECT), whereas the former is the tracer of choice for MIBG therapy. [^123^I]-MIBG scintigraphy has a sensitivity of 77–90% and a specificity of 95–100% in the detection of PPGLs [15]. The highly diverse sensitivity can partially be explained by the variable expression of the NET depending on the differentiation and the genetics of the tumors, and on the use of planar scintigraphy *versus* SPECT (more sensitive). Mechanistically, it has been demonstrated that in SDHx-mutated tumors, which are more often metastatic, the accumulation of oncometabolites inhibiting ten–eleven translocation (TET) methylcytosine dioxygenases induces hypermethylation and suppression of gene expression [38]. Among the genes downregulated in these tumors is *SLC6A2*, encoding the NET. The Cancer Genome Atlas (TCGA) PPGL dataset confirmed that there are some patients of the pseudohypoxic cluster showing low levels of *SLC6A2* (Fig. 3). Although for these patients imaging targeting the NET might not be effective, for the majority of PPGL patients such approach should be appropriate. Noteworthy, therapy using the electron emitting radionuclide [^131^I-MIBG] is the only currently approved therapy for unresectable/metastatic PPGLs with MIBG-positive lesions.

One of the drawbacks of [^123^I]-MIBG scintigraphy is the limitation in staging and restaging of hereditary and metastatic PPGLs [15], with an especially poor performance in detecting SDHx-mutated tumors. Therefore, novel approaches aimed developing PET tracers, or at increasing the therapeutic radioactive dose, have been tested in small animal models.

A new synthesis method has allowed to produce [^18^F]- (for diagnosis) or [^131^I]-labeled (for therapy) (4-fluoro-3-iodobenzyl)guanidine (FIBG), a derivative of MIBG [39]. To evaluate this novel tracer, biodistribution studies, PET imaging and a therapeutic study were performed on PC12-derived mouse xenografts. [^18^F]-FIBG showed good tumor-to-background ratio, with lower incubations times than the original [^131^I]-MIBG. [^131^I]-FIBG was retained in higher amounts and for longer time in the tumors when compared with MIBG, and this was associated with a stronger inhibition of tumor growth following a therapeutic dose of [^131^I]-FIBG versus [^131^I]-MIBG. Interestingly, the uptake of [^18^F]-FIBG in the xenografts was proportional to the therapeutic effect of [^131^I]-FIBG. Thus, this tracer could be used in a theranostic approach, both for the detection ([^18^F]- labeled) and for the therapy ([^131^I]- labeled) of PPGLs.

#### Positron emission tomography (PET)

Nuclear medicine uses radiotracers, radioactively labeled ligands at very low (picomolar to nanomolar) concentrations, to visualize functional processes. Several radiotracers have been developed for PPGLS, which take advantage of the expression of specific receptors and transport systems on the membrane of chromaffin cells (Fig. 1).

#### Imaging the NET with meta-[^18^F]fluorobenzylguanidine ([^18^F]-MFBG)

As outlined above, [^123^I]-MIBG only allows for planar and SPECT imaging, which has a lower sensitivity and poorer spatial resolution compared with PET. Other disadvantages include the need for a 2-day protocol and thyroid blockade. ^18^F-meta-fluorobenzylguanidine ([^18^F]-MFBG) is a promising alternative for [^123^I]-MIBG that offers one-day, high-resolution PET/MR imaging. In pilot trials of patients with neural crest tumors (including PPGL), [^18^F]-MFBG imaging was safe, had favorable biodistribution and kinetics with good targeting of lesions [40]. In preclinical studies on neuroblastoma xenografts, [^18^F]-MFBG showed a slightly lower lesion uptake compared with [^123^I]-MIBG, but had significantly faster blood clearance thus enabling high-contrast visualization of lesions already 1 h after injection [41]. This tracer has yet to be evaluated in PPGL models.

#### Imaging the NET with [^18^F]-6-fluoro-dopamine ([^18^F-FDA])

Among the agents available to localize PPGLs is [^18^F]-6-fluoro-dopamine ([^18^F-FDA]), which is also internalized into chromaffin cells by NET. In the study of Martiniova and colleagues mentioned above, a comparison of [^18^F]-DOPA and [^18^F]-FDA was done in parallel in the MPC metastatic model. [^18^F]-FDA showed a lower uptake compared to [^18^F]-DOPA and detected fewer metastatic lesions. A few years later, the same group compared [^18^F]-DOPA and [^18^F]-FDA for the detection of subcutaneous or metastatic MPC-derived tumors in mice [42]. They observed a lower accumulation of [^18^F]-FDA compared to [^18^F]-DOPA in large liver lesions, presumably due to a decrease in NET expression following tumor dedifferentiation. Interestingly, when MPC cells were injected subcutaneously, only [^18^F]-DOPA PET, but not [^18^F]-FDA could detect the tumors and this may reflect the expression of VMAT 1 and VMAT2 in the tumor cells (Fig. 1) [42]. This data is in conflict with the results obtained in human patients, where [^18^F]-DOPA had indeed a high sensitivity (81%-100%) in detecting non-metastatic PPGLs [43]. Yet, the sensitivity in metastatic cases was as low as 45% and even lower in SDHB-related cases. In contrast, [^18^F]-FDA PET/CT showed equally good sensitivity for localizing primary and metastatic PPGLs (77% versus 76%), also in patients with metastatic SDHB-mutated tumors [43]. Thus, the MPC metastatic model may not precisely mimic the situation observed in the patients. Yet, studying the differences in uptake of these two radiopharmaceuticals in these models may shed light into the molecular mechanisms leading to the differences in functional imaging outputs observed in clinical imaging of the various human PPGL subtypes. Moreover, the direct comparison of different tracers would be highly relevant in xenograft/allograft models of genetically modified PPGL cells belonging to other subclusters, given that MPC cells harboring an Nf1 mutation represent the kinase-signaling cluster.

#### Imaging the LAT Transporter with [^18^F]-dihydroxyphenylalanine ([^18^F]-DOPA)

One of the ligands that can be used to target chromaffin cells is dihydroxyphenylalanine (DOPA), which is transported into catecholamine-secreting tumors by the large amino acid transporter (LAT). Once internalized, DOPA is converted to dopamine by the aromatic amino acid decarboxylase and stored in vesicular granules (Fig. 1). The tracer [^18^F]-DOPA has been widely used for the detection of human PPGLs by PET imaging [44] and shows 84–100% sensitivity and 88–100% specificity [33]. One limitation is, however, that PPGLs with a non-secretory phenotype might not express the LAT transporter, thereby leading to a limited [^18^F]-DOPA accumulation [45].

By combining MRI and [^18^F]-DOPA, further improvements to the imaging of both the subcutaneous and the metastatic MPC-derived mouse models (see above) were achieved. Indeed, Martiniova and colleagues showed that tumor lesions in several organs could be readily detected by [^18^F]-DOPA PET/MRI [22]. In a subsequent study on human PPGL patients, the combination of [^18^F]-DOPA PET with MRI was found to be superior to MRI-alone and also able to achieve a higher diagnostic confidence than [^18^F]-DOPA PET/CT [46].

#### Somatostatin receptor imaging

Somatostatin is a neurotransmitter regulating the release of hormones by various target organs including the pituitary gland (growth hormone), pancreas (insulin, glucagon) and the gastrointestinal tract (gastrin). It demonstrates anti-proliferative and anti-angiogenic effects on cells expressing somatostatin receptors (SSTRs). The broad expression of SSTRs in neuroendocrine tumors led to the development of several somatostatin analogs (SSAs) for diagnosis and therapy. There are five SSTR subtypes (SSTR1-5) with variable expression levels according to the tissue type and having different affinities to the clinically available SSAs. PPGLs express mostly SSTR2 and SSTR3 at a lower level, while SSTR1, SSTR4 and SSTR5 are usually absent, as an immunohistochemical study on 151 PPGL patients revealed [47].

Among the most common imaging modality to visualize neuroendocrine tumors is [^111^In]-pentetreotide scintigraphy (Octreoscan). This tracer has the highest affinity for SSTR2. In clinical practice, emission data (SPECT) are fused with CT data to improve diagnostic accuracy. Due to suboptimal image quality and relatively high effective doses, a derivative of Octreoscan was created using ^99m^Tc, resulting in the tracer [^99m^Tc]-HYNIC-TOC (Fig. 1). Introducing the positron emitter ^68^Ga to label SSAs allowed the application of these tracers to PET imaging. Among the available radiolabeled SSAs, three should be especially emphasized: [^68^Ga]-DOTATATE and [^68^Ga]-DOTA-TOC (^68^Ga-DOTA-d-Phe1-Tyr3-octreotide), [^68^Ga]-DOTA-NOC: DOTA-1-Nal3-octreotide (for more information on SSTR-directed imaging read Patel et al. [48]). The minor chemical modifications on the SSAs affect their affinity to the different SSTR subtypes (illustrated in Table 1), thereby allowing to select the imaging tracer according to the relative levels of the various SSTRs in the tumors. [^68^Ga]-SSA PET can overcome the low resolution of both scintigraphy and conventional SPECT imaging, and is especially useful for the detection of extra-adrenal PPGLs, where it shows higher sensitivity than [^123^I]-MIBG [12]. Noteworthy, [^68^Ga]-SSA PET has superior sensitivity at detecting SDHx-mutated PPGLs than [18F]-FDG-PET [49].

**Table 1:** Specificity of the SSA ligands toward the SSTR receptors. Depicted are in vitro affinity profiles (50% inhibitory concentration (IC50) in nM ± standard[52]

	SSTR1	SSTR2	SSTR3	SSTR4	SSTR5
^68^ Ga-DOTATATE	> 10,000	*0.2 ± 0.04*	*> 1000*	300 ± 140	377 ± 18
^68^ Ga-DOTA-TOC	> 10,000	*2.5 ± 0.5*	*613 ± 140*	> 1000	73 ± 12
^68^ Ga-DOTA-NOC	> 10,000	*1.9 ± 0.4*	*40 ± 5.8*	260 ± 74	7.2 ± 1.6

Additionally, replacement of ^68^Ga with the radioisotopes ^177^Lu or ^90^Y allows to use these SSAs for targeted peptide receptor radionuclide therapy (PRRT). PRRT represents the current approved indication in Europe and in the USA or patients with advanced (metastatic) and/or progressive neuroendocrine tumors positive on somatostatin receptor imaging, who cannot undergo surgery and whose symptoms do not respond to other medical therapies. [^177^Lu]-DOTATATE-based PRRT has been evaluated in patients with unresectable, locally advanced or metastatic PPGL and has emerged as a viable alternative to [^131^I]-MIBG [50, 51].

Both [^68^Ga]-DOTATATE and [^68^Ga]-DOTA-TOC tracers were used in a preclinical study from Ullrich et al. 2018, to detect MPC cells labeled with firefly luciferase (luc) and engrafted in various immunocompromised mouse strains [53]. Besides demonstrating that SCID/beige and SKH1 mice are the best suited host strains for MPC-derived metastases studies, the authors also showed that *Sstr2* expression was maintained in MPC cells-derived metastases and could be well detected by using the two radiolabeled SSAs. Unfortunately, a direct comparison of the SSAs in the same animals was not performed. These studies established Sstr-directed imaging as a modality suitable to monitor the response of this metastatic PPGL allograft model to therapies.

#### Imaging glucose metabolism with [^18^F]-fluoro-D-glucose ([^18^F]^−^FDG)-PET

[^18^F]-FDG represents the most often applied PET imaging tracer and is widely used in many cancer entities. It is based on the high demand of nutrients by cancer cells to support their growth. Uptake of radiolabeled glucose is therefore higher in metabolically active cells, including tumor cells. [^18^F]-FDG uptake is variable in PPGLs depending on their genetic background, degree of differentiation and malignancy [54, 55]. [^18^F]-FDG is particularly recommended for patients with known metastatic PPGLs or with mutations in SDHB (at higher risk for metastases) [43]. Indeed, a hallmark of pseudohypoxic PPGLs (often associated with SDHx mutations) is their shift from oxidative phosphorylation to aerobic glycolysis (the so-called Warburg effect). Consequently, these tumors show a rapid glucose turnover. Therefore, [^18^F]-FDG PET may be used to obtain an in vivo metabolic tumor profiling in patients with PPGLs belonging to the pseudohypoxic cluster [56]. Additionally, and in agreement with what has been reported for other solid tumors, [^18^F]-FDG PET could be of value in monitoring early treatment response, as tumor cell metabolism adapts much faster than any other parameter detected by standard tumor markers (i.e., cell growth, cell death, tumor vascularization) [57].

**[**^18^F-FDG] PET was recently evaluated by Facchin et al., 2020, as part of a novel trimodal imaging system that combined anatomical imaging with X-ray CT, metabolic imaging with [^18^F]-FDG PET and ultrafast ultrasound imaging (UII) [58]. This imaging sequence referred to as PETRUS (PET Registered Ultrafast Sonography). Ultrasound is usually not recommended for diagnosis of PPGLs, however it was included in the PETRUS sequence in order to image vascular network changes. Specifically, PETRUS was employed to follow the response to the multikinase inhibitor sunitinib of an allograft model obtained by injecting immortalized mouse *Sdhb*-/- cells. This drug inhibits the vascular endothelial growth factor receptor (VEGFR), RET and other kinases. VEGFA is the most important pro-angiogenic factor and as such is upregulated in many tumors. PPGLs are highly vascularized and have a high expression of VEGFA especially in malignant and large tumors. Thus, they are expected to be sensitive to inhibitors of VEGFRs. Therefore, sunitinib was tested on patients with progressive PPGLs in a Phase II clinical study, which was recently completed [27]. In contrast to the expectations, the overall effect of sunitinib in this clinical study was low. Although sunitinib treatment is not beneficial in most PPGL patients, it could be effective in a subset of them, i.e., those showing pseudohypoxic features, known to induce neoangiogenesis, or patients with RET mutations, another receptor kinase targeted by sunitinib. The PETRUS imaging scheme could beautifully monitor the response to sunitinib in the *Sdhb*-/- PPGL allograft model. Although ultrasound is generally only used for the detection of PPGL if CT scans cannot be performed, the authors used it to measure several parameters related to vascularization. Interestingly, following an initial response, the *Sdhb*-/- PPGL allografts developed resistance to sunitinib treatment after a few weeks. The mechanism of resistance might be interesting to follow up on as it could shed light on the low efficacy of this drug against human PPGLs. The PETRUS imaging approach appears to be an interesting imaging modality, suitable to monitor the response of PPGLs to drugs targeting tumor vascularization.

#### Usage of radiosensitizers to improve radionucleotide therapy and imaging

Radiosensitizers are highly relevant in the cancer field given their ability to enhance the efficacy of radiation therapy. Current radiosensitizers increase the lethal effect of radiotherapy on tumor cells by inhibiting the radiation-induced DNA damage response or improve the local uptake of radionuclides by upregulating the expression of the corresponding target receptors on the cancer cells. The latter strategy could be suitable to improve imaging of malignant, dedifferentiated PPGLs with downregulated expression of NET. Histone deacetylase (HDAC) inhibitors act as epigenetic modulators as well as post-transcriptional regulators of acetylated proteins and were found to restore chromatin accessibility and thereby gene expression. In the MPC-derived metastatic mouse model of PPGLs, [^123^I]-MIBG and [^18^F]-FDA uptake by the tumors could be enhanced by a pretreatment with the HDAC inhibitor romidepsin or trichostatin A thanks to enhanced receptor expression and retention of the radioisotopes [59]. As also suggested by the authors, radiosensitization might profit significantly from further preclinical studies analyzing biodistribution and kinetics of receptor expression, to then infer the optimal timepoint for drug administration. Various animal models able to recapitulate the features of dedifferentiated tumors (of the different subclusters) would be extremely helpful to tackle the problem of the imaging sensitivity drop observed in the most aggressive cases.

#### Bioluminescence and fluorescence imaging

Bioluminescence and fluorescence imaging are highly sensitive methods with a long history in tumor imaging in small animals. They rely on the use of genetically modified (labeled) cell lines engrafted into mice. In order to follow PPGL progression longitudinally in live animals, Ullrich et al. subcutaneously injected mCherry-labeled MPC cells into nude mice and performed whole-body fluorescence imaging to monitor tumor development [60]. Tumors in this allograft model could be easily detected and quantified by combining fluorescence and MRI imaging approaches. Additional measurements of urinary free monoamines allowed the correlation of fluorescence signal intensities with a functional tumor marker [60]. As tumor growth (by caliper measurements) had a positive linear relationship with fluorescence intensity and monoamine secretion, these parameters emerged as useful markers to follow tumor progression, and hold promise as readouts to monitor the response of PPGL allografts to drug treatment in vivo.

### Preclinical studies with novel tracers

#### Fluorescence imaging of Cox-2 tracer

The greatest strength of animal models is the possibility to test novel imaging techniques that, if promising, might then be translated to the patients. A potential novel target for the detection of PPGLs is the enzyme Cox-2, which is involved in the synthesis of prostaglandin. Cox-2 expression is upregulated in several tumors, and its activity associates with promotion of angiogenesis, invasion of tumor cells and resistance to chemotherapy [61]. Cox-2 inhibitors have been shown to improve sensitivity of tumor cells to radiation [62]. Several studies found an increased expression of Cox-2 in aggressive PPGLs and suggested this enzyme as a marker to discriminate benign from malignant tumors [63–65]. As MPC-luc cells used to generate the preclinical allograft PPGL model described above [60] also express Cox-2, a fluorescently labeled Cox-2 tracer was evaluated in this model and the results compared to bioluminescence imaging [66]. The XenoLight RediJect COX-2 probe accumulated in the tumors (yet, a strong and unspecific tracer accumulation at injection site was also detectable). Thus, a Cox-2-directed tracer might be valuable in assessing the expression level of the enzyme in view of treatment with Cox-2 inhibitors to trigger PPGLs radiosensitization. A Cox-2-targeting tracer for PET imaging has been developed and tested in a colorectal cancer xenograft model [67], but has not yet been applied to PPGLs.

### Imaging of endogenously developing PCCs in animal models—examples of MENX-associated PCCs

The previously mentioned imaging studies used allograft or xenograft models of PPGL cell lines to monitor tumor growth, as well as to detect lesions in live animals. However, there are some limitations: subcutaneous tumor models do not reproduce the tumor microenvironment of PPGLs; and metastatic MPC-derived models (by intravenous injection) have very high tumor multiplicity, and the aggressiveness of the disease leads to termination of the studies after only few weeks.

Among the few available animal models of endogenous PPGLs is the MENX rat strain. Tumor formation in this model is caused by a spontaneous frameshift mutation in *Cdkn1b*, encoding the cell cycle regulator p27kip1 (p27). This mutation associates with a loss of function of p27 and with uncontrolled cell proliferation [68]. Homozygous mutant rats develop bilateral PCC (frequency 100%) with a clear progression from hyperplasia (age 4–5 months) to full-blown PCCs (age 8–10 months). They also present with abdominal PGLs (frequency 10–20%)[69]. Recently, we showed that rat PCCs show pseudohypoxic features [70]. These studies established MENX rats as the only currently available model of spontaneous, endogenous pseudohypoxic PCCs. The kinetics of tumor development in this model allow the analysis of the various stages of progression, in contrast to the fast-growing cell lines used to generate xenograft/allograft models. Endogenous tumors in MENX rats are therefore highly valuable to evaluate imaging modalities and to perform preclinical therapy studies in view of a clinical translation. With this in mind, several imaging modalities used to visualize PPGLs in patients were tested in MENX rats to demonstrate whether this model resembles the human disease concerning the uptake of specific tracers (see also Fig. 2).

#### Comparison of [^11^C]-HED and [^68^Ga]-DOTATOC

The uptake of ^11^C-hydroxyephedrine ([^11^C]-HED), targeting the NET, and [^68^Ga-DOTATOC, targeting SSTR receptors, were compared side by side in 8–10-month-old MENX rats (bearing histologically confirmed tumors) and control unaffected rats [71]. Tracer uptake was correlated with the expression of *Sstr* genes and of *Slc6a2* encoding the NET. Both tracers showed a stronger uptake in the adrenal glands of mutant rats compared with unaffected adrenals of wild-type animals. However, ^11^C-HED/PET provided better signal-to-noise ratio compared to ^68^Ga-DOTATOC. While *Sstr2* mRNA levels were significantly upregulated in rat PCCs *versus* normal adrenal, the expression of *Slc6a2* was, on average, not differentially regulated (although some individual tumors exhibited a higher expression). The superior performance of ^11^C-HED/PET suggests that there could be a different post-transcriptional regulation of *Slc6a2* in tumor versus normal medullary cells leading to higher expression of the NET in the former. Furthermore, by using ^68^Ga-DOTATOC, there was a high background signal originating from the kidneys that made the detection of the adrenal glands difficult. In a retrospective study of 134 PPGL patients imaged with ^11^C-HED/PET and PET/CT, a sensitivity of 91% and a specificity of 100% of the former could be revealed. Interestingly, ^11^C-HED/PET imaging was less sensitive in MEN2 patients [72]. Although, ^11^C-HED provides excellent results in the detection of PCC, it is rarely used in human patients: the 20-min half-life of the tracer requires on-site synthesis, which is available only in few specialized centers.

#### Comparison of ^18^F-LMI1195 and ^123^I-MIBG

**Fig. 4:**
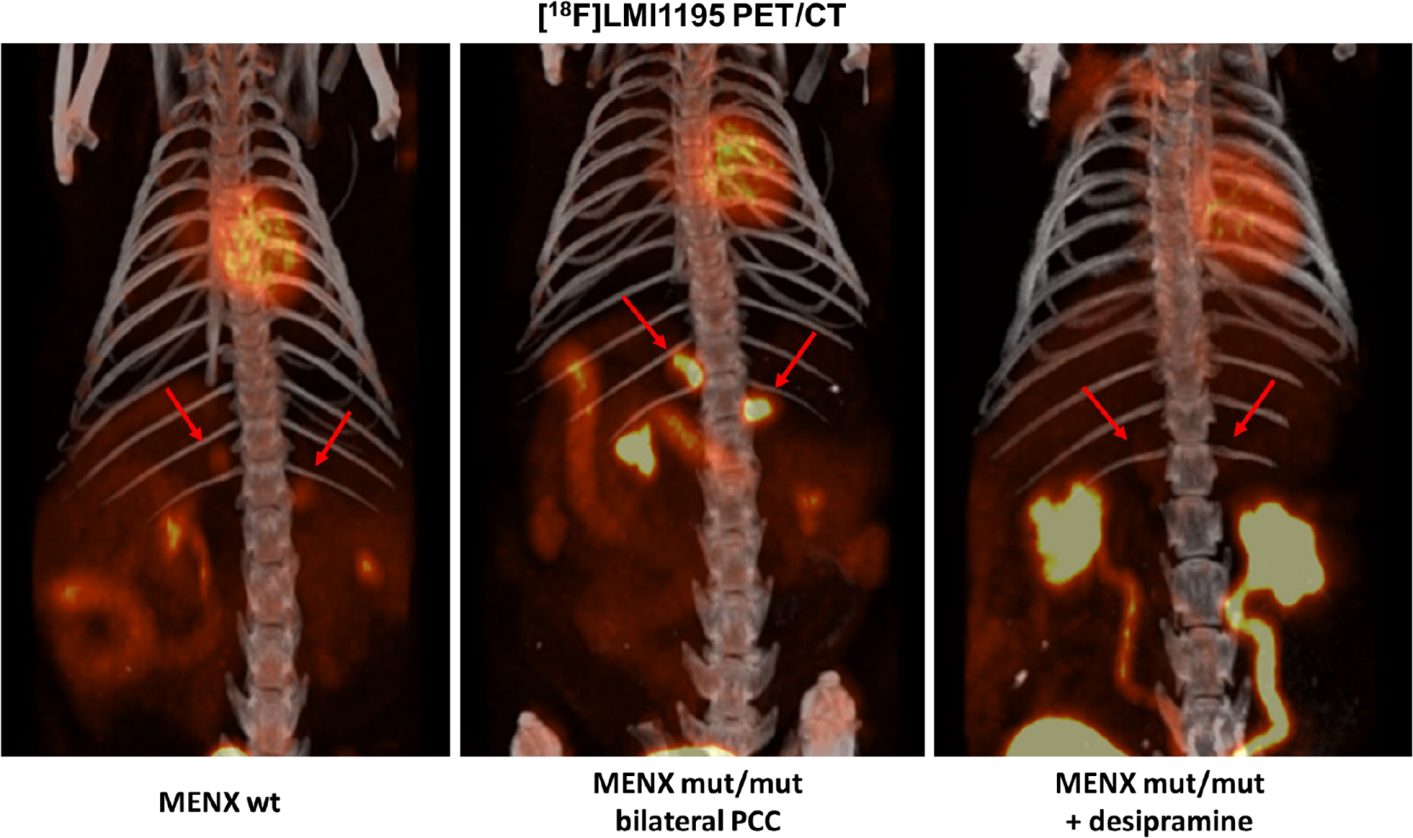
PCC imaging with [^18^F]-LMI1195 PET/CT in MENX rats. Exemplary pictures of [18F]-LMI1195 composite PET/CT images. While in normal adrenal glands of a MENX wild-type rat, there is only a moderate accumulation of the tracer (left). An intense tracer accumulation is however observed in adrenal glands of PCC-bearing mut/mut rat (middle). Despiramine, which blocks the uptake of norepinephrine via the NET transporter, reduces also the uptake of the tracer in the PCCs (right)

In 2013, we showed that the catecholamine analog, [^18^F]-LMI1195 (also known as [^18^F]-Flubrobenguane) is a promising novel PET tracer for the detection of PCCs in MENX-affected rats [73]. This tracer is a structural analog of [^123^I]-MIBG and targets the NET encoded by *Slc6a2*. Imaging by [^18^F]-PET has the advantage of higher spatial and temporal resolution along with better attenuation correction, absolute quantification and shorter acquisition times than [^123^I]-based SPECT/CT [74]. [^18^F] offers the same benefits as [^11^C] without the disadvantage of its short half-life. In comparison with its predecessor [^18^F]-FIBG, [^18^F]-LMI1195 offers the advantage that it is not degraded by monoamine oxidase, and it can be more easily synthesized than [^18^F]-FDA [74]. When [^18^F]-LMI1195 was injected into wildtype rats the maximum signal peak in adrenals could be observed after 1 min but a high signal was kept over 60 min, when other background signal in other organs had already washed out [73]. The adrenals of PCC-bearing MENX rats showed much higher standardized uptake value (SUV) compared to normal adrenals of wildtype rats, despite having a similar background noise signal in other organs. The [^18^F]-LMI1195 signal could be blocked by despiramine, a selective norepinephrine reuptake inhibitor (Fig. 4). Noteworthy, in this study, [^123^I]-MIBG and [^18^F]-LMI1195 were directly compared against each other in the same MENX rats. While the signal of both tracers was equally good at detecting tumor-bearing adrenals, the background signal was lower for [^18^F]-LMI1195 in organs such as liver, lung, small intestine and bone, a feature that could be relevant for the imaging of PPGL metastases or small primary tumors.

After this preclinical proof-of-concept study in the MENX rats and a phase I study in healthy humans assessing human safety, whole-organ biodistribution and radiation dosimetry [75], [^18^F]-LMI1195 was evaluated in a retrospective study of 24 patients with suspected PPGLs. Patients scans were performed between 2016 and 2018, and the data were recently published [76]. The highest SUV could be observed in thyroid, pancreas and tumor lesions, with unaffected adrenal glands and liver parenchyma having significantly lower SUV compared to the tumors. The intense accumulation of [^18^F]-LMI1195 in thyroid and pancreas is not unexpected, given these organs have extensive sympathetic innervation where NET is expressed. Surgery and subsequent histological analysis of 13 patients revealed that 11 of them were correctly diagnosed: 9 with PCC and 2 with suspected PGL. The hybrid detection of [^18^F]-LMI1195 PET and CT/MRI detected more and smaller lesions than conventional imaging, but this did not reach statistical significance probably due to the low patient number. However, diagnosis confidence and detection size was better in [^18^F]-LMI1195 PET/CT/MRI than CT/MRI alone [76]. The data strongly speaks for further evaluation of this tracer in larger patient cohorts and a comparison with the gold standard MIBG SPECT/CT.

While targeting NET has its limitations for PPGL imaging, which also applies to [^18^F]-LMI1195, namely the loss of receptor expression in some PPGL tumors, this novel tracer has the potential to be superior to the standard clinical practice. This study represents a prime example of how diagnosis of PPGL patients could be improved by using novel tracers originally tested in a suitable preclinical animal model.

#### Multispectral optoacoustic tomography

An emerging optical imaging method is multispectral optoacoustic tomography (MSOT) where fast tunable lasers excite tissues with short light impulses of multiple wavelengths. The resulting thermal tissue expansion induces a photoecho, ultrasound waves that can be subsequently measured. This technique provides noninvasive, high-resolution tomographic images of absorbers such as oxygenated and deoxygenated hemoglobin, lipids and melanin [77]. As such, MSOT is particularly indicated to image tissue vascularization in vivo without the need of contrast agents. Furthermore, MSOT can be also combined with imaging probes, as quantum dots, GFP or gold particles to visualize biomolecules and their tissue distribution [78]. The maximum penetration depth of 2–3 cm in tissues limits at the moment its application to the diagnosis of PPGLs, but it may be highly suitable to image the progression of xenograft tumors.

As mentioned earlier, PPGLs, especially those with high HIF expression (pseudohypoxic cluster), are highly vascularized tumors, with chaotic vessel architecture [79]. In a proof-of-principle study, we investigated whether MSOT might be used to study PPGL vascularization in vivo. Unfortunately, the size of the rats does not allow direct imaging using the preclinical MSOT scanner. Therefore, as experimental model, we used xenografts of primary rat PCC cells. These cells represent a more physiological system than established cell lines. Moreover, rat primary cells derive from pseudohypoxic tumors (in humans, the most aggressive cluster), whereas MPC cells used for the allograft studies have a kinase activation profile. Primary rat PCCs were successfully engrafted subcutaneously in nude mice, and oxygen saturation was longitudinally monitored by MSOT imaging during tumor growth [70]. The results showed that the tumor grafts derived from the rat PCC cells have high levels of oxygenated hemoglobin (Fig. 2). Thus, this approach might be exploited in the future to monitor the response of pseudohypoxic PCC cells to anti-angiogenic drugs in vivo. Recent studies have demonstrated the high quality of the vessel resolution by MSOT in breast cancer and its suitability to monitor drug treatment response [80], thereby suggesting that this modality might be applicable to preclinical PPGL research as well.

#### Ex vivo biological imaging of intact PCC vasculature

Recent advances in sample preparation techniques to increase optical transparency of tissue specimens have boosted the use of biological imaging using of light sheet fluorescence microscope systems (LSFM) and broadened its applicability in biological research. An important application of LSFM is the visualization of whole vascular networks in intact organs or tumors in model organisms. While this technique does not allow to follow neoangiogenesis over time, it provides images of intact organs with the highest possible resolution. To investigate the vasculature architecture of rat PCCs, MENX rats endothelial cells were labeled using a fluorescent T-lectin probe and the adrenal glands collected and cleared for LSFM analysis ex vivo. The results revealed the high vessel density and chaotic vessel formation in MENX PCCs (Fig. 2) [70].

Further improvements, such as the novel deep learning-based metastasize analysis in cleared tissues (DeepMACT), should allow the most precise detection of metastasis in animal models at single cell level [81]. While the resolution of DeepMACT is unbeatable, it does not allow to monitor therapy response over time. For this, PET/CT, using tracers that have a high specificity and a good signal-to-noise ratio in order to discriminate tumor from normal tissue, is still the preferred method.

## Conclusion

Accurate diagnosis of PPGLs is essential given the comorbidities associated with undiagnosed/untreated tumors. Moreover, their functional characterization may guide targeted therapies and radionuclide therapy. Medical imaging plays a critical role in both these aspects. Preclinical imaging studies with animal models of PPGLs helped assessing their faithfulness as models of the cognate human tumors. Several imaging modalities, from microCT to functional PET, led to high-resolution detection of PPGLs in xenograft/allograft and in endogenous rodent models. For example, MPC-derived metastases in allograft models could be detected with microCT and [^68^Ga]-SSA PET. The possibility to detect PPGLs and their metastases with high sensitivity in preclinical settings is a prerequisite to exploit these models for therapy studies. Along these lines, the multimodal PETRUS sequence used by Facchin et al. [58] proved to be suitable to monitor the response of PPGL allografts to anti-angiogenic drugs and could be translated in clinical practice. As a prototypical example of the translational value of in vivo PPGL models, the initial evaluation of a novel PET tracer in MENX rats set the basis for the application of LMI1195/Flubrobenguane in patients with suspected PPGLs. This tracer emerged as useful tool for the staging of PPGLs and now awaits validation in larger patient cohorts.

## Abbreviations

CT: Computed tomography
MRI: Magnetic resonance imaging
MTT: Mouse tumor tissue cell line
NET: Norepinephrine transporter
PCC: Pheochromocytoma
PDX: Patient-derived xenograft
PET: Positron emission tomography
PPGL: Pheochromocytoma and paraganglioma
SPECT: Single-photon positron emission tomography

## Declarations

### Availability of data and materials

All data generated or analyzed during this study are included in this published article.
